# Regulation of Energy Expenditure and Brown/Beige Thermogenic Activity by Interleukins: New Roles for Old Actors

**DOI:** 10.3390/ijms19092569

**Published:** 2018-08-29

**Authors:** María del Carmen García, Patricia Pazos, Luis Lima, Carlos Diéguez

**Affiliations:** 1 Department of Physiology/Research Center of Molecular Medicine and Chronic Diseases (CIMUS), University of Santiago de Compostela, 15782 Santiago de Compostela, Spain; conscity@hotmail.com (P.P.); luis.lima@usc.es (L.L.); carlos.dieguez@usc.es (C.D.); 2 Instituto de Investigación Sanitaria de Santiago de Compostela (IDIS), 15706 Santiago de Compostela, Spain; 3 CIBER Fisiopatología Obesidad y Nutrición (CB06/03), Instituto de Salud Carlos III (ISCIII, Ministerio de Economía y Competitividad (MINECO)), C/Monforte de Lemos 3-5, Pabellón 11. Planta 0, 28029 Madrid, Spain

**Keywords:** cytokines, interleukins, inflammation, energy and metabolic homeostasis, thermogenesis, brown and beige adipose tissue

## Abstract

Obesity rates and the burden of metabolic associated diseases are escalating worldwide Energy burning brown and inducible beige adipocytes in human adipose tissues (ATs) have attracted considerable attention due to their therapeutic potential to counteract the deleterious metabolic effects of nutritional overload and overweight. Recent research has highlighted the relevance of resident and recruited ATs immune cell populations and their signalling mediators, cytokines, as modulators of the thermogenic activity of brown and beige ATs. In this review, we first provide an overview of the developmental, cellular and functional heterogeneity of the AT organ, as well as reported molecular switches of its heat-producing machinery. We also discuss the key contribution of various interleukins signalling pathways to energy and metabolic homeostasis and their roles in the biogenesis and function of brown and beige adipocytes. Besides local actions, attention is also drawn to their influence in the central nervous system (CNS) networks governing energy expenditure.

## 1. Introduction

The obesity epidemic is worsening worldwide, particularly among youths and young adults [1].Consequently, serious challenges will impact the health care systems in the near future: A progressively earlier onset of obesity associated chronic diseases such as type 2 diabetes mellitus (T2DM), fatty liver, cardiovascular and chronic kidney disease, as well as neurodegenerative disorders and cancer; and a proportional increase in the morbidity load into middle age [2]. Decades of research ground the notion that localized immune cell infiltration in white adipose tissue (WAT), driven by the energy-surplus in obesity, promotes a low grade systemic inflammation which in turn induces a global impairment of insulin action. Metabolic derangements related to obesity are largely mediated by insulin resistance (IR), which greatly increases the risk of T2DM and the burden of its co-morbidities [3,4].

Nevertheless, there might be some beneficial effects of WAT-related immune responses. As in other major metabolic organs, inflammation and inflammatory mediators generated by resident immune cell populations and stromal cells, play essential roles in the maintenance of tissue integrity by stimulating its healthy expansion, remodelling and even repair [4,5,6]. Immune surveillance also extends to local energy and nutrient availability, thereby influencing the metabolic and endocrine performance of adipocytes to meet the metabolic needs derived from over nutrition [4]. Increased adipocyte secretion of various hormones, such as leptin, triggers a brain feed-back-loop that reduces food intake and activates sympathetic nervous system (SNS). This acute adaptive mechanism counteracts the anabolic pressure of increased insulin secretion through increases in the rate of lipolysis and thermogenic processes [5]. However, resolution is needed and during conditions of sustained positive energy balance, this otherwise physiological response, is perpetuated and become pathogenic. Altered production of several cytokines, adipokines and lipid species, as well as activation of multiple immune receptors and intracellular mediators, have been associated with insulin and catecholamine resistance leading to overall metabolic disruption [5,7,8,9,10]. However, metabolic homeostasis still requires an active immune system, since WAT disruption of inflammatory pathways leads to adipocyte dysfunction, dysbiosis and chronic systemic inflammation, as seen in obesity [4,11]. Thus, duration and magnitude of immune responses are key outcome determinants. In line, many so-called pro and anti-inflammatory molecules have been shown to exert contradictory dose and time dependent actions, also influenced by their production and target sites [4]. This also seems to be the case of the classical brown (BAT) and recently discovered beige adipose tissues [12].

In response to cold, brown and, to a lesser extent, beige adipose cells (also called “brite” adipocytes), have the capacity to burn fat or glucose to release energy in the form of heat, in a process called non-shivering thermogenesis (NST) [13]. Their somewhat shared morphological and functional properties are mainly related to the presence of multilocular lipid droplets and a high content of mitochondria expressing uncoupling protein 1 (UCP1) [14]. Recruitment of beige adipocytes in rodent WAT—also termed “WAT browning” or “beiging”—is an adaptive and reversible response to environmental stimuli including: chronic cold acclimation, exercise and nutritional challenges; as well as external and internal cues such as: pharmacological treatment with β3-adrenergic receptor (AR) agonists or thiazolidinediones (TZDs) and various peptides and hormones [12,15]. Indeed, both white and brown fat pads also contain innate immune cells, including M2-like macrophages, eosinophils and innate lymphoid type 2 cells (ILC2s), acting as positive actors in the control of BAT thermogenic activity and WAT browning. Though not exempt of controversies, recent research suggests that a balanced Type 2/Type 1 inflammatory response is essential to maintain the integrity and hormonal sensitivity of brown and beige adipocytes or their precursor cells and regulate sympathetic innervation of thermogenic adipose tissue (AT) [12,16,17,18].

Despite initial controversies about prevalence of BAT in adult humans [19,20], cumulative evidence supports its relevance and the existence of inducible beige-like thermogenic adipocytes that significantly contribute to the regulation of systemic energy homeostasis [13,21]. Constitutive BAT activity is inversely correlated with adiposity, blood glucose concentrations and insulin sensitivity [21,22]. Meanwhile, chronic cold acclimation promotes the recruitment of new thermogenic fat even in subjects with undetectable levels of pre-existing BAT, as proven by Positron Emission Tomography/Computerized Tomography (PET/CT) studies [23,24,25,26]. Interestingly, a substantial proportion of adult BAT located in the neck and supraclavicular region shows a gene expression pattern selective to mouse beige adipocytes [27]; while the deep neck regions resemble classical brown fat in mice [13,28]. Cold inducible-BAT activity correlates with increases in NST and/or an improvement in insulin sensitivity [24,25]. Thereby, fat browning has gained considerable attention due to is potential as a new therapeutic target in the treatment of obesity and its metabolic co-morbidities. However, this conclusion should be viewed with caution since detrimental effects linked to overactive browning activity have been recently identified as main pathogenic substrate in inflammatory hypermetabolic conditions, such as cancer cachexia and burn injury [29,30,31].

This review aims to summarize and discuss evidence from genetic and pharmacological interventions in rodents (Table 1), as well as human studies reporting beneficial or deleterious effects of various cytokines on energy expenditure (EE) through beige and brown fat activation. Besides local actions, we will draw attention to their influence in the central nervous system (CNS) networks governing, through hypothalamic mediated SNS efferences, the thermoregulatory and metabolically driven alterations in BAT and beige thermogenesis.

## 2. The Scenery: Natural History of Thermogenic and Immune Cells in Adipose Tissues

### 2.1. Commitment and Differentiation of Thermogenic Adipocytes

During embryogenesis and adult life, adipose tissue formation, hyperplasia and cell turnover is supported by active proliferation and differentiation of adipocyte precursor cells into new mature adipocytes [13,32]. Consistent with the postnatal relevance of NST in thermoregulation, brown adipocytes differentiate from cell progenitors and constitute mature BAT during mouse embryonic stages (precursors commitment stage at embryonic day E12.5 and preadipocyte stage at E14). Indeed, morphologically differentiated BAT pads are easily distinguished by haematoxylin-eosin staining and perilipin (mature adipocyte marker) immunohistochemistry by E15.5 and rapidly expand until birth [33,34]. Despite, the first WAT adipocyte precursors and subsequent cell lineage commitment are detected at E10.5 and E18, subcutaneous (scWAT) and visceral WAT (vWAT) development and maturity proceed at neonatal and postnatal stages [35]. Earlier microarray and lineage-tracing studies identified a subpopulation of dermomyotomes marked with a specific transcription factor gene expression profile (*PAX7*, engrailed 1 (*EN1*); and myogenic factor 5 (*MYF5*)-positive), as shared ancestors between brown adipocytes and muscle cells but not with any white adipocyte (MYF5-negative) [36,37]. Yet, gene expression of early B cell factor 2 (*EBF2*, a marker of brown preadipocytes) and myoblast determination protein (*MYOD*, a muscle specific transcription factor) are detected in separate cell populations of the E12.5 somitic mesoderm [34]. In fact, when expressed ectopically in myoblasts, EBF2 induces brown adipogenesis by repressing the expression of MYOD and another muscle-specific transcription factor, myogenin [38]. Additional determinants of the brown adipose fate by silencing and activating respectively the myogenic and the BAT gene programme comprise, among others, two members of the PR Domain Zinc Finger family of proteins (PRDM3 and PRDM16) and its crucial enzymatic activator, the histone methyltransferase EHMT1. Lack of *EHMT1* leads to rudimentary brown fat formation in mice and its interaction with either PRDM3 or PRDM16 is essential to trigger brown adipogenesis [39]. Interestingly, *PRDM16* deletion in MYF5^+^ precursors does not affect embryonic BAT development but, suggesting a compensatory action, PRDM3 leads to disrupted BAT identity and function in adulthood, which was accelerated to the postnatal stage by simultaneous *PRDM16* deletion [40].

Meanwhile, the embryonic origin of white and beige adipocyte progenitors residing in mature adipose tissues, appears to be more complex than expected [35,41]. Recent evidences [42,43] suggest that a PAX3^+^ subset of MYF5^+^ progenitor cells are also capable to undergo white and/or beige adipogenesis in specific fat depots (retroperitoneal, anterior subcutaneous and, male but not female, gonadal WAT). Further pointing toward progenitor heterogeneity among different fat depots, genders and ages. Indeed, analyses of clonal adipogenic progenitor cell lines indicate that divergences already exist between beige and white fat precursors, showing distinctive distribution and molecular profiles in mice [27] and adult humans [44,45], with only a few enriched markers in common. Among these, the tyrosine kinases platelet-derived growth factor receptors α and β (PDGFRA and B) have recently been proposed as selective makers for precursor lineages committed later to beige (PDGFRA) or white (PDGFRB) adipogenesis during AT development and adulthood [46]. Other markers of early beige progenitor cells also include: myosin heavy chain 11 (MYH11^+^, from smooth muscle cells progenitors) or stem cells antigen 1 (SCA1^+^) [47].

The thermogenic competency gene program is activated in the mesenchymal precursors in response to various endocrine cues such as bone morphogenetic proteins (BMP) and fibroblast growth factors (FGFs) [48,49]. Several members of these signalling protein families appear to play a key role not only in brown and beige differentiation but also in their functional activation, including: BMP4, 6, 7 and 8B, as well as FGF16, 19 and 21. For instance, ablation of BMP signalling, by either MYF5^+^ progenitor cells specific knockout of *BMP receptor 1A* [50] or *BMP7* global deletion [51], leads to a severe paucity of classical BAT at embryonic and adult stages in mice. Conversely, BMP7 can also induce differentiation of human adipose stem cells to beige adipocytes [52], so does BMP6 and to a lesser extent BMP7, on human and murine muscle precursors to brown cells [53].

Besides the above mentioned, to date studies have identified roughly 50 transcriptional and epigenetic activators/repressors of brown and beige adipocyte differentiation (reviewed in detail in [47]). Of note, most such factors operate through four transcriptional regulators, namely: Peroxisome Proliferator-Activated Receptor Γ (PPARG), CCAAT/Enhancer Binding Protein β (CEBPB), Peroxisome Proliferator-Activated Receptor Γ Coactivator 1-α (PPARGC1A) and PRDM16. While both PPARG and CEBPB bind DNA regions directly, PRDM16 uses its zinc Finger domains to form a transcriptional complex with these and other transcription factors and therefore stipulates and activates the BAT selective gene program [37,54,55]. BAT-associated genes include among others those coding for: the uncoupler UCP1, its inhibitor CIDEA (Cell death-Inducing DFFA-like effector A) [56], the cytochrome oxidase subunits COX7A1 and COX8B, the fatty acid elongase ELOVL3 and the FFA mitochondrial carrier (Carnitine Palmitoyl-Transferase 1 A) CPT1A [57]. The transcriptional-coactivator PPARGC1A is a key regulator of mitochondrial biogenesis and expression of genes involved in: free fatty acid (FFA) transport and oxidation, oxidative phosphorylation (OXPHOS) and thermogenesis in differentiated brown and beige adipocytes [58,59]. For instance, PPARGC1A co-activates nuclear respiratory factors 1 and 2 (NRF1 and NRF2), with the consequent overexpression of the mitochondrial transcription factor A (*TFAM*), which in turn activates transcription and replication of the mitochondrial genome [60]. In addition, PPARGC1A interacts with PPARG and peroxisome proliferator-activated receptor α (PPARA) to jointly or independently stimulate the transcription of *UCP1* and lipid uptake and metabolism related genes such as: FFA binding proteins (*FABPs*), *CPT1*, acyl-CoA dehydrogenases *LCAD* and *MCAD* [61,62,63]. Thus, chronic treatment with PPARG agonists (TZDs) strongly induces browning of WAT through PRDM16 stabilization [47]. However, results from a recent study shows that pharmacological PPARA activation or *PPARA* deletion have a minimal or null effect on expression of cold-induced genes in murine WAT, arguing against a relevant role for this factor in beige adipocyte activation in this setting [64].

Despite the scarce knowledge regarding their cellular lineage specification, beige adipocytes develop postnatally in response to a number of environmental and internal stimuli such as chronic cold exposure, exercise, pharmacological interventions and several endocrine hormones (reviewed in detail in [65]). Most of the known transcriptional and epigenetic regulators of brown adipogenesis exert similar functions in beige development. As in classical BAT precursors, EBF2 is highly enriched in PDGFRA^+^ progenitor cells [34] and promotes beige biogenesis in mouse scWAT [66]. Of note, PRDM16 by its own or in association with other chromatin modifying enzymes, the carboxy-terminal binding proteins (CTBP1 and CTBP2), acts as major activator of the beige fat program [67]. Though PRDM16 brown pre-adipocyte specific depletion has minimal effects on their differentiation and function, it severely impairs beige adipocyte formation in response to cold and β 3 sympathetic agonists [68]. Last but not least, upon stimuli, beige adipocyte can also emerge within adipose tissues through trans differentiation of mature unilocular UCP1 negative white adipocytes [14]

### 2.2. Regulation of Thermogenic Activity

The hallmark of brown and beige adipocytes is their thermogenic capacity, which mostly relies on the mitochondrial abundance and activity of the NST protein mediator, UCP1. This inner mitochondrial membrane protein stimulates thermogenesis by dissipating the proton gradient generated by electron transport in the respiratory chain because of substrate oxidation and thereby the energy needed for ATP synthesis [69]. Notably, the paradoxical metabolic and thermoregulatory phenotypes of BAT or beige depleted mice and UCP1 deficient mice (reviewed in [13]), suggested the existence of UCP1-independent thermogenic mechanisms. Recent studies have reported several protein or substrate mediated uncoupling pathways in brown or beige fat inducing NST, including: SERCA2b-mediated Ca^2+^ cycling, creatine-driven substrate cycle, peptidase M20 domain containing 1 (PM20D1) induction of lipidated amino acid uncouplers, or the activation of ATP/ADP mitochondrial translocators (ANT) [70,71,72,73]. Though the crucial role of UCP1 in BAT thermogenesis is well appreciated, future research is warranted to clarify the relative contribution and coordination of canonical (UCP1) and non-canonical (UCP1 independent) AT heat generation to whole-body energy and metabolic homeostasis in different conditions. Of note, new methods to monitor thermogenic fat activity in vivo and in cultured cells, assaying not only canonical activation but also various non-UCP1-mediated thermogenic pathways, have recently been developed [32,74].

In line with the above discussed, the thermogenic program in ATs is not limited to the transcriptional induction and activation of UCP1, complex metabolic adaptations also occur, including enhanced mitochondrial biogenesis [60], aimed at increase fuel supply and oxidation [75,76,77,78,79]. The turn-on of the brown/beige thermogenic “circuitry” can be achieved through various “switches” [65,80,81]: (1) CNS activation modulating sympathetic output to ATs [82]; (2) recruitment and activation of immune cells in ATs (discussed in the following section) [12]; (3) direct action of circulating factors and hormones on adipocytes or beige precursor cells [15] and (4) local crosstalk between these cells and the vasculature through secretion of angiogenic factors (mainly vascular endothelial growth factor A (VEGFA)) and subsequent increased nutrient and oxygen supply [83,84,85]. As previously mentioned, various external cues unlock these switches (cold exposure, exercise, nutritional challenges), this is particularly important given the inducible nature and activity of beige adipocytes and its relevance in human physiology. Among these, here we will briefly describe the canonical hypothalamic-SNS-AT pathway increasing BAT thermogenic activity and WAT browning in response to cold.

The hypothalamic preoptic area (POA) is the primary central sensor involved in the maintenance of body temperature homeostasis integrating external and internal temperature input from central and peripheral thermoreceptors. Cold-activated POA neurons synapse in other nuclei within the hypothalamus to form complex connections (reviewed in detail in [82]) and subsequently project to the rostral raphe nucleus (rPPa) in the brain stem, leading to an increase sympathetic traffic to BAT and WAT. Catecholamine release in ATs nerve terminal endings actives G-protein coupled receptors and thereby lipolysis through increased cyclic AMP (cAMP) and activation of protein kinase A (PKA) or Src-dependent activation of extracellular signal regulated kinases (ERK1/2) [86]. Activated PKA in turn phosphorylates, among other intracellular mediators, p38 mitogen activated protein kinase (p38 MAPK) and CRE-binding protein (CREB) [63,87], leading to increased expression and activation of PPARGC1A.

### 2.3. Immune Cells in Brown and Beige Adipose Tissue

The basic physiological unit of all metabolic organs, including ATs, is formed by the combo of parenchymal, stromal and immune cells [17]. During development and adulthood, each element of this combo is devoted to specialized roles in a temporally and spatially coordinated manner to maintain overall energy, metabolic and tissue homeostasis. As previously discussed, white, beige and brown cells are respectively responsible to store excess of nutrients and generate heat from the use of their metabolic derivatives in uncoupled respiration. Parallel functions of stromal and immune cells include to surveil and improve their performance in response to environmental (cold, nutritional overload or restriction, exercise) and developmental challenges. To do so, the heterogeneous stromal cell populations in WAT depots (discussed in Section 2.1 and reviewed in [13,17,35,47]): (1) support tissue expansion and cell turnover needs through differentiation; (2) ensure tissue structural integrity and (3) orchestrate adipogenesis, as well as recruitment and activation of immune cells through their secreted proteins. These effector molecules include: extracellular matrix proteins, growth factors (insulin, macrophage colony and granulocyte-macrophage colony stimulating factors (IGF1, CSF-1 and GM-CSF)), chemokines and cytokines.

The immune combo element in WAT is primarily constituted by macrophages, ILC2s, eosinophils and regulatory T cells (Tregs) (for recent reviews see [12,16,88,89]). Adipose tissue macrophages (ATMs) display a multifaceted nature due to their ability to adopt a range of pro- and anti-inflammatory phenotypes in response to environmental cues, in a process known as polarization [90]. The common designations classically and alternative activated or M1-pro and M2-anti-inflammatory originally derived from in vitro stimulation studies [90,91]. Thus, the M1 type related to bacterial pathogen elimination, expressed high levels of Type 1 cytokines (e.g., tumour necrosis factor (TNF) and interleukin 1 (IL1)) and inducible nitric oxide synthase (iNOS) in response to interferon γ (IFNG) and/or lipopolysaccharide (LPS). Conversely, the M2 type involved in parasite containment and tissue remodelling, were characterized by the IL4 induced gene expression of anti-inflammatory proteins including Ym1, arginase 1 (*ARG1*) and type 2 cytokines such as *IL10*. Thereby, this M1 and M2 terminology was used to generally depict the proinflammatory state of largely recruited ATMs in obesity versus the predominant anti-inflammatory state of resident ATMs in leanness [92]. However, this paradigm does not exactly resemble the in vivo setting, where it is likely that a polarization spectrum of subpopulations exits within ATMs, from the most pro-inflammatory to anti-inflammatory state. Indeed, in obesity infiltrated ATMs display a slightly different phenotype from that seen in macrophage-mediated immune responses to infection. These so-called metabolic activated macrophages (MMe) are stimulated by glucose, insulin and saturated FAs to produce inflammatory cytokines and to overexpress lipid metabolism but not classic M1 cell surface markers [93]. The subsequent recruitment and activation of other immune cells such as neutrophils, CD8^+^ and CD4^+^ T cells and natural killer cells (NK) leads to the overall type 1 immune phenotype and sustained inflammation in obesity [12,16]. The M2-like phenotype of resident ATMs is instead sustained by sensors of FFAs and their derivatives such as PPARG [17], adipose-derived mesenchymal stem cells [94] and type 2 cytokines secreted by the immune network formed by ILC2s and eosinophils (discussed in the following sections). Research over the last decade have grounded the consideration of M2 like macrophages and type 2 signalling as positive actors in the control of glucose and lipid metabolism in WAT, by ameliorating inflammation and insulin resistance and promoting the browning process under different settings [12]. In 2011 Nguyen et al. [95] reported that cold exposure quickly induced a M2-like phenotype in brown ATMs leading to increased BAT thermogenic activity and also lipolysis in WAT. This finding was somewhat unexpected, given the scarce knowledge at that time regarding the identity of BAT resident immune populations and the controversial envisioning of cold-activated macrophages as an additional source of catecholamines sustaining NST. Following studies by Rao et al. [96] and Qiu et al. [97] provided further support to those findings. In summary, these reports showed that in response to cold exposure the subsequent adipocyte secretion of meteorin-like hormone (METRNL) triggered eosinophilia in sc WAT, which in turn could induce tissue browning by increased release of Type 2 cytokines and ATMs recruitment and activation. Other external cues such as caloric restriction [98], exercise [96] and microbiota depletion [99] have been reported to enhance functional beige fat in obese mice through activation of this innate immune cell network, leading to significant improvements of systemic metabolism.

The contribution of ILC2s, as the third step of this innate type 2 cell axis, by modulating beige adipogenesis and growth, was recently described by Brestoff et al. [100] and Lee et al. [101] in almost simultaneous publications. In the first study [100], activated ILC2s-derived methionine-encephalin peptides were shown to directly stimulate the differentiation and activation of beige adipocytes. In the second one, a two-step mechanism was proposed: While ILC2s and eosinophil cooperated to promote the expansion and commitment of adipocyte precursors to the beige fat lineage, myeloid cell-derived catecholamines were responsible of their conversion in mature functional cells. In line with this proposal, activated epididymal WAT resident ILC2s were previously shown to facilitate local accumulation of eosinophils and anti-inflammatory polarization of ATMs [102]. However, this connection between ILC2s-eosinophils and macrophages to increase AT catecholamine levels is currently a subject of great debate in the field (nicely reviewed in [12,16]). Finally, other resident immune cell populations, such as Tregs [103] or invariant natural killer T (iNKT) cells [104], have been shown to sustain or enhance brown and beige thermogenic function by limiting inflammation or secreting the thermogenic inducer FGF1.

## 3. Cytokines: Immune Actors in BAT Activation and Browning of WAT

**Figure 1:**
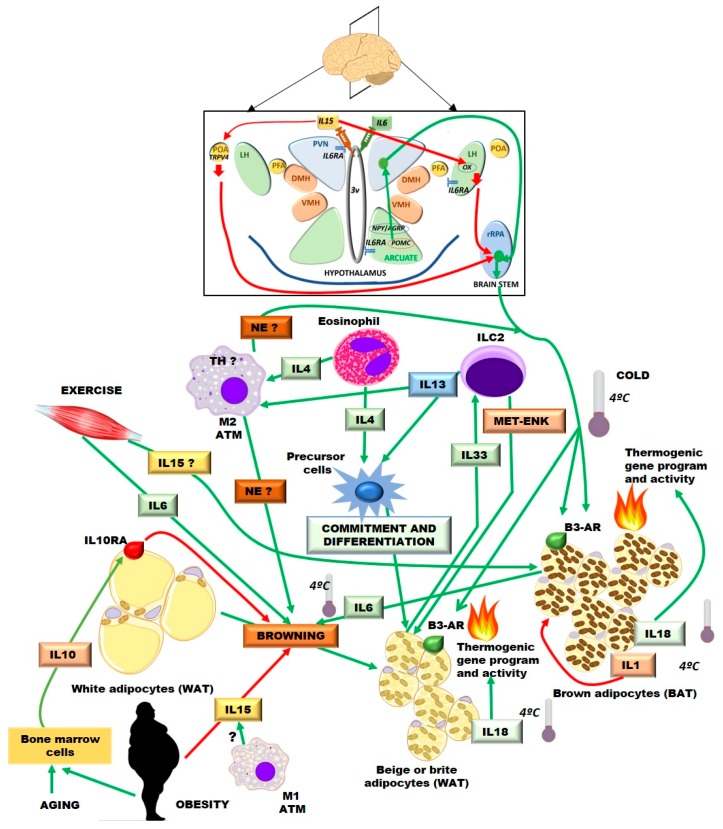
Main interleukins involved in the modulation of EE and BAT and beige adipose tissue thermogenesis through CNS or direct actions in brown adipocytes activation and WAT browning. In case of available data, tissue and cell sources of this immune mediators are also shown. Red or green lines and arrows depict inhibitory or stimulatory actions. Inset in the upper panel represents a scheme of the main hypothalamic and brain stem nucleus involved in the central regulation of ATs thermogenic function, namely: preoptic area (POA), dorsomedial (DMH), arcuate (ARC), paraventricular (PVH) and lateral nucleus of the hypothalamus (LH) as well as the rostral raphe nucleus (rRPA) at the brain stem. Hypothalamic mediators at these nuclei include: agouti-related protein (AgRP), neuropeptide Y (NPY) and proopiomelanocortin (POMC) at the ARC, orexin at the LH and transient vanilloid rector 4 (TRPV4) at the POA. IL6 and IL15 are known to activate or inhibit these central pathways. rRPA receives input from the different hypothalamic areas to activate sympathetic projections to ATs acting at β3 adrenoceptors (B3-AR). Some interleukins are directly produced by resident or recruited immune cells, including: adipose tissue macrophages (ATMs, M2-alternative and M1-classically activated), eosinophils and type 2 innate lymphoid cells (ILC2) to stimulate WAT browning through commitment and differentiation of precursor cells at this site. The ability of M2-ATMs to produce catecholamines (tyrosine hydroxylase, TH) to sustain adaptive thermogenesis is under debate. Several stimuli have been reported to trigger or inhibit both mechanisms including among others stimulation by cold exposure and exercise, while others such as obesity and aging exert the opposite effect. Question marks depict mechanisms unknown or under debate. Methionine-enkephalin (MET-ENK).

Scene coordination in this complex play is possible thanks to several signalling molecules and metabolites. Actors, secreted and sensed by the combo members, including: chemokines, cytokines, hormones and growth factors but also FAs, glucose and amino-acids and its derivatives. In this section, we will try to briefly summarize the roles of different cytokines, mainly interleukins, with reported central or peripheral actions in BAT and beige thermogenesis (Figure 1).

### 3.1. IL4 and IL13, to Be or Not Be: Eosinophils, Macrophages and the Catecolaminergic Controversy

#### 3.1.1. IL4/IL13 Production and Signalling

IL4 and IL13, the closely related canonical type 2 cytokines, belong to the short-chain four-helix bundle cytokine family with about 25% sequence similarity [105]. Aside to their classical inhibitory role on type 1 inflammation (INFG, IL12 and NO), IL4/IL13 initiate potent type 2 inflammatory responses including among others Th2 T cells differentiation and M2 macrophage polarization. Though they share receptor subunits and signalling pathways, these two cytokines mediate common but also unique physiological effects [106]. The divergence in actions is related to segregation in their cellular and tissue sources and exclusive receptor binding subunits. Both cytokines are secreted similarly by Th2-polarized T cells, granulocytes and monocytes/macrophages, while mice iNKT2 cells and ILC2s and human CRTH2^+^ type 2 ILC are important sources of IL13 but only secrete IL4 under certain circumstances [106]. Though at low levels, the ubiquitous expression of their common ligand binding receptor subunits (*α*, *IL4RA* (Gene ID: 16190) and *α1*, *IL13RA1* (Gene ID: 16164)) in most non-immune cell types turn them into IL4/IL13 potential targets. Conversely, the common γ signalling subunit (*IL2RG* Gene ID: 16186) is shared with other cytokine receptor complexes and primarily expressed on hematopoietic immune cells.

After binding of IL4 with IL4RA, two possible heterodimer receptor complexes are formed by recruitment of IL2RG (type I) or IL13RA1 chains (type II), leading respectively to JAKs activation of signal transducer and activator of transcription 6 (STAT6) and insulin receptor substrate 2 (IRS2) or predominantly STAT6. Thereby the availability of each chain in the cell membrane determines the selected type of receptor [107]. Instead, IL13 binds with IL13RA with lower affinity than the complex IL4:IL4RA and subsequently recruits IL14RA to further signalling trough JAKs/STAT6 but also STAT1 and 3 [106,108]. Hence, competition for IL13RA recruitment between IL4: IL4RA and IL13 due to different concentrations of the cytokines in the extracellular milieu might determine the functional outcomes [105].

IL4/IL13 signalling is not only involved in modulation of the immune responses but also in mammary development and lactation [109], as well as higher functions of the normal brain, such as memory and learning [110]. Conversely, their actions on energy and metabolic homeostasis appear to be, especially for IL4 [111] but not as clearly for IL13 [108], at the crossroads between inflammation and nutrient metabolism.

#### 3.1.2. IL4/IL13 and Energy and Metabolic Homeostasis

First evidences for an IL4/IL13 regulatory role in systemic metabolism indicated that STAT6, the common signalling intermediate, was expressed in mouse liver and WAT and activated by in vivo and in vitro IL4 treatment only in hepatocytes [111]. Moreover, metabolic reliance of IL4/STAT6 stimulated hepatocytes on glucose oxidation was mediated by repression of PPARA transcriptional regulation of key genes for β- and ω-oxidation of FAs. Conversely, *STAT6* null mice were resistant to diet induced obesity (DIO) due to enhanced EE, associated with PPARA driven FA oxidative metabolism in the liver and lipolysis and browning (measured by *PGC1B* and *UCP1* expression) in WAT. Despite this lean phenotype, high fat diet (HFD)-fed *STAT6* KO mice developed hepatic insulin resistance and steatosis. Strikingly, IL4 peripheral treatment during HFD feeding in mice reduced weight gain and adiposity due to increased locomotor activity-related EE. Additionally, an overall improvement of insulin sensitivity was observed in association with a significant inhibition of the PPARA-regulated catabolic program in liver and increased expression of M2-like macrophage markers in WAT. However, the opposite effect was reported to occur when IL4 was administered intracerebroventricularly (icv) to rats during HFD feeding, that is, exacerbation of obesity, further hypothalamic inflammation as well as leptin and insulin resistance [112]. Of note, these central obesogenic effects occur independently of caloric intake, pointing to decreased EE or altered substrate metabolism as leading cause. Overall these results suggested that beneficial metabolic effects of the IL4/STAT6 axis were peripherally exerted and related, at least in part, to its anti-inflammatory properties.

On the contrary, IL13 has been reported to directly target hepatic glucose production through a non-canonical downstream pathway—IL13RA1/STAT3—and beyond its modulatory role in inflammation [108,113]. Thus, IL13 deficient mice showed increased body weight, caused by a decreased EE, dislipemia, fasting hyperglycaemia and hyperinsulinemia as aged [108]. Dysregulated glucose metabolism, in this model was due to insulin resistance in major metabolic organs (WAT, muscle and especially liver), enhanced hepatic glucose production and steatosis. Blunted fast to feed inhibition of hepatic gluconeogenic gene expression was also observed in young *IL13* KO mice preceding disturbances in serum lipid, fasting insulin and glucose levels. Gain or loss of function experiments in primary hepatocytes revealed that this effect was mediated by IL13RA1 activation of STAT3. Of note, minimal or even null expression changes were observed in WAT or BAT of M2-like or classical M1 macrophage activation or mitochondrial oxidative metabolism and thermogenesis gene markers. Overall, these results indicated that the inhibitory action of IL13 on hepatic glucose productions were beyond its immunomodulatory roles.

#### 3.1.3. IL4/IL13 and the Thermogenic Function of Adipose Tissues

As discussed above, both IL4 and IL13 are potent inducers of Type 2 inflammatory responses including macrophage polarization and acute cold exposure was found to induce specifically BAT and WAT gene expression of alternative activation markers such as: *ARG1*, mannose receptor C-1 (MRC1) and C-type lectin domain containing 10A (*CLEC10A1*) [95]. In line with these findings, in the same publication Qiu et al. demonstrated that mice with global *IL4*/*IL13* or *STAT6* and *IL4RA* myeloid cell-specific deletion but also with pharmacological depletion of macrophages (clodronate-containing liposomes-treated) were more prone to acute cold-induced hypothermia. These phenotypes were accompanied with blunted cold-activation of thermogenic gene program (as measured by mRNA levels of *UCP1*, *PPARGA* and the FA oxidase *ACOX1*) and gene expression of M2-like markers in BAT. Mechanistically, this pro-thermogenic effect of alternative activated ATMs was proposed to be related to their ability to synthetize and secrete norepinephrine (NE) in response to IL4 through induction of catecholamine-synthesizing enzymes including tyrosine hydroxylase (TH). Subsequent work of the same group [97] and others showed a similar recruitment of M2-like macrophages into sc WAT in response to cold and other environmental [96,98,114] or internal cues [94,99,115,116,117,118,119]. Additionally, new cellular and molecular elements or regulators of this so-called fat thermogenic circuit or axis were identified: (1) eosinophils as source of IL4 [96,97,120], (2) ILC2s producing IL13 to stimulate beige adipocyte precursors proliferation and commitment [101], (3) adipose or muscle derived METRLN as trigger of IL4 production by eosinophils [96] and (4) adiponectin as direct inducer of M2-like ATMs proliferation through its binding partner T-cadherin [121]. Depletion of each of these cells or molecular mediators blunted browning of scWAT. However, this paradigm has been recently challenged by various publications disputing a significant role of M2-like macrophages and especially of IL4-ATMs mediated adrenergic activation of BAT and beige thermogenesis [122,123,124,125,126]. The core of the debate is supported by a multi-centre study which thoroughly shows evidences against a substantial capacity of alternative ATMs to produce and secrete catecholamines [124]. Thus, adoptive transfer of *TH* conditional deleted bone marrow-derived cells (BMDCs) into irradiated mice showed that, unlike peripheral deletion of this enzyme, specific lack in macrophages did not affect overall EE, core temperature and BAT thermogenic gene expression in response to cold. Additionally, treatment of sc WAT and BAT primary cells with conditioned media from IL4-stimulated BMDMs caused a robust macrophage polarization but failed to induce thermogenesis in adipocytes. Finally, IL4 treatment to BMDS did not enhance NE secretion, as measured by HPLC of the culture media. Immunohistochemistry or flow cytometry and RNA sequencing analysis failed to detect cold induced-TH expression in M2-like ATMs. Overall these results strongly support the notion that ATMs cannot produce catecholamines to induce BAT activation and WAT browning but also cast doubts regarding the upstream role of eosinophils in this thermogenic axis. Indeed, Bolus et al. [127] recently reported that increasing vWAT eosinophilia in HFD fed mice by IL5 (eosinophils survival and proliferation activator) treatment to the levels observed in chow reared controls did not counteract obesity, IR or systemic inflammation. Moreover, in the same report cold challenge for 48h further increased eosinophil vWAT content in these mice but no indication of increased EE, browning or M2-like recruitment were observed in this fat pad depot.

However recent studies have shed light into this controversy. Firstly, a subpopulation of macrophages lying near AT sympathetic nerve endings, termed sympathetic nerve associated macrophages (SAMs), have showed capacity to uptake NE through the SLC6A2 transporter and catabolize NE trough monoamine oxidase A [126]. SAMs increase in number with age and impair lipolysis in AT by tittering the local level of catecholamines [128]. Specific deletion of *SLC6A2* in SAMs protects mice against DIO by increasing thermogenesis in BAT and scWAT. Since analysis of polarization markers were not performed, given the reciprocal activation/repression between pro and anti-inflammatory ATMs populations, it was proposed that a low M2-like/high-M1-like phenotype will be compatible with the actions of SAMs in NE turnover [12]. Secondly, resident ATMs in BAT have been recently shown to participate in the long-term control of sympathetic tissue innervation through the repressive action of the transcription factor methyl-CpG-binding protein 2 (*MECP2*) on the expression of receptor *PlexinA4* [129]. Specific deletion of *MECP2* in macrophages in mice impairs local NE signalling, *UCP1* expression and BAT thermogenesis, leading to adulthood obesity. BAT overexpression of Plexin 4 in this mouse model contributes to specifically repel of *Sema6A*-expressing sympathetic axons. Thirdly, catecholamine or UCP1-independent mechanisms [94,130,131,132] have been proposed supporting a role of ATMs in BAT and beige thermogenic functions. Overall, these results imply that, yet through an unclear mechanism, macrophages can shape browning responses in WAT and the acute or long-term activity of classical BAT.

### 3.2. IL33, an ILC2-Derived Perinatal BAT Licenser and Adult Beige Fat Activator of Thermogenesis

#### 3.2.1. IL33 Production and Signalling

IL33, is a member of the IL1 family of cytokines, constitutively expressed in the nucleus of endothelial, epithelial and fibroblast-like cells (Gene accession number: ID: 77125), both during homeostasis and inflammation [133]. IL33 was postulated as an “alarmin”, a protein released in the extracellular space after cellular stress or mechanical injury to alert the immune system of threatens to cell and tissue integrity. Various stimuli have been shown to trigger IL33 secretion such as: infectious agents, allergens, trauma and cellular necrosis [134]. Upon transient secretion, the nuclear full-length protein IL33FL is biologically active but cleavage by proteases from neutrophils and mast cells renders highly active mature forms of the cytokine [133,134]. Both the released and cleaved forms of IL33 critically modulate the functions of their major target cells: mast cells, ILC2s and Tregs, that constitutively express the IL33 receptor termed ST2 or IL1RL1 [135,136]. However, IL33 can elicit type-2 but also type-1, immune responses trough inducible *ST2* expression in other immune cells such as: macrophages, NK, Th1 and CD8^+^ T cells [133]. Of note, other IL33 non-immune targets, constitutively expressing ST2, have been described, including adipocytes, preadipocytes, endothelial cells, epithelial cells, fibroblasts, astrocytes and neurons; expanding the potential roles of this cytokine in homeostatic and disease conditions [133,137,138].

Close similarity exists between signalling pathways activated by IL33 and other members of the IL1 cytokine family: IL1 and IL18. Once IL33 binds to ST2 membrane receptor, a complex is formed with a shared co-receptor between members of this cytokine family, the IL1 receptor accessory protein (IL1RAP). Subsequently, the IL33/ST2/IL1RAP complex triggers the MyD88/IRAK1-4 (IL1 receptor associated kinases 1 and 4)/TRAF6 intracellular signalling pathway, ultimately leading to activation of AP1 and NFKB transcription factors [136]. Thereby, differences in the biological activity of IL1, IL18 and IL33 are related to expression of their specific receptors in target cells. Finally, a soluble form of ST2 (sST2), which acts as a decoy receptor, blocks IL33/ST2 signalling by sequestering free IL33 [139].

#### 3.2.2. IL33 and Energy and Metabolic Homeostasis

Over the last decade, murine studies have provided evidence of a protective effect of IL33 on systemic metabolism [16,139]. IL33 in vitro treatment of differentiating adipocytes induced IL5 and IL13 production, decreased expression of adipokines and genes associated with adipogenesis and lipid metabolism and inhibited lipid accumulation [140]. IL33 in vivo treatment in genetically (*ob*/*ob*) or DIO mice caused significant reductions in fat mass and fasting glucose levels, as well as ameliorated insulin and glucose tolerance along with accumulation of M2-like ATMs and ST2^+^ Tregs in vWAT [140,141]. Conversely, *ST2* null mice were more prone to develop DIO and glucose overload-induced hyperglycaemia than their WT counterparts [140]. WAT Tregs are considered as crucial actors in metabolic syndrome development [142]. Obesity in humans is associated with decreased circulating Tregs levels, which were inversely correlated with body weight and BMI [143]. Given their immunomodulatory role, it was considered that fat Tregs might reduce adipose inflammation, promoting overall metabolic homeostasis. Indeed, reduction of WAT Tregs populations in mice by *PPARG* specific deletion in these cells was accompanied with marked inflammatory response in vWAT [144]. DIO in mice is also associated with drastically reduced WAT Treg numbers, which were reverted by treatment with the PPARG agonist pioglitazone as were glucose intolerance and insulin resistance in WT mice but not in mice harbouring PPARG-deficient Tregs [144]. Overall, these results suggest that IL33/ST2 might counteract obesity IR through its proliferative actions on Tregs. Indeed, IL33 and ST2 are also expressed in human WAT [138] and population-based studies have found positive correlations between increased serum levels of the IL33 soluble decoy receptor (sST2) and obesity, T2DM and their metabolic complications [145,146,147,148].

#### 3.2.3. IL33 and Thermogenic Function of Adipose Tissues

As previously discussed there are other major immune cell targets of IL33 actions: mast cells and specially ILC2s. Four articles to date [100,101,149,150] have described a role of the IL33/ST2/ILC2s axis on the WAT browning process and BAT activation through different mechanisms. First, Brestoff et al. [100] provided evidences that obesity in mice and humans is characterized by decreased ILC2s populations in WAT, an effect also seen in IL33 deficient mice. The early onset-obese phenotype of *IL33* null mice was also associated with increased adiposity and dysregulated glucose homeostasis. Conversely, IL33 treatment prevented DIO in mice by increasing EE and WAT browning, in association with increased WAT ILC2s numbers and improved glucose homeostasis. Notably, IL33-induced recruitment of beige adipocytes was only dependent of ILC2s activation, as adoptive transfer of these cells was sufficient to drive browning independently of eosinophils and IL4R activation of ATMs. Indeed, authors identified IL33 activated-ILC2s as a source of methionine-encephalin peptides with the ability to directly activate beige adipogenesis and the thermogenic program (e.g., *UCP1* gene expression) in scWAT. On the contrary, Lee et al. [101], Hams et al. [149] and Ding et al. [150] using different experimental approaches proposed a scenario in which IL33 activated-ILC2s led to IL5 mediated eosinophil activation and subsequent IL4-mediated macrophage polarization and beige fat biogenesis and activation. Additional complexity was added when it was reported [151] that the IL33/ST2 system, through yet undefined mechanisms is essential to activation of uncoupled mitochondrial respiration during perinatal stages in mice, rendering *IL33* null mice cold intolerant due to abnormal splicing of *UCP1*.

### 3.3. IL6, Central and Peripheral Modulator of AT Thermogenesis

#### 3.3.1. IL6 Production and Signalling

IL6 is a cornerstone cytokine with a broad spectrum of biological functions in both health and disease states [152,153]. Consistently, this pleiotropic cytokine can be produced and secreted under appropriate stimuli by several immune and non-immune cell types in different tissues (Gene ID: 16193) including: monocytes, macrophages, astrocytes, B and T cells but also fibroblasts, endothelial and skeletal muscle cells, neurons, adipocytes and hepatocytes [154,155,156,157]. Almost all inflammatory and infectious processes and types of cancer are associated with a strong induction of circulating IL6 levels [158,159,160] but also in a lesser extent various non-pathological conditions such as exercise [161] and pregnancy [162]. The plethora of pro- and anti-inflammatory actions of this cytokine is supported by the ubiquitous expression of the signal-transducing subunit of its receptor, the protein gp130 and the context and tissue dependent-balance between its classical and trans-signalling pathways [158]. IL6 shares gp130 with other members of this cytokines family such as: ciliary neurotrophic factor (CNTF), leukaemia inhibitory factor (LIF), oncostatin M (OSM), cardiotrophin 1 (CT-1), cardiotrophin-like cytokine (CLC) and IL 11 and 27 [163] but displays an exclusive receptor IL6RA with ligand recognition but not signalling properties. Unlike *gp130*, *IL6RA* shows a restricted expression pattern (Gene ID: 16194), being found both peripherally and at the CNS in only a few cell types including: hepatocytes, some leukocytes and epithelial cells but also astrocytes, neurons and glial cells such as tanycytes [155,157,158,164,165,166]. Classical signalling occurs after binding of the IL6/IL6RA complex to membrane anchored gp130 through the phosphorylation of downstream targets such as JAK/STAT, ERK and PI3 kinase. Notably, a soluble form of IL6RA also exists (sIL6R), generated by its limited proteolysis and less frequently by alternative gene splicing. IL6 shows similar affinity for both membrane and soluble receptors and the complex formed by sIL6R/IL6 also binds to gp130 and subsequently induces dimerization and activates intracellular signalling pathways. This process termed trans-signalling is of pivotal importance, since it renders non-IL6RA expressing cells IL6-responsive. Over the last decade a myriad of research publications has helped to decipher the contributions of both pathways to the IL6 biological actions (thoroughly summarized in excellent reviews [153,155,157,164]).

#### 3.3.2. IL6 and Energy and Metabolic Homeostasis

As a key immune mediator of the low-grade chronic inflammation in obesity, elevated serum IL6 and CRP levels are characteristically found in obese patients [167] and considered as risk factors for T2DM development [168]. Indeed, increased fat mass and infiltrated macrophages are closely related to the systemic IL6 rise in T2DM patients [169] and its concomitant reduction with weight loss was associated with IR amelioration [170]. However, anabolic side effects of anti-IL6 therapies are commonly reported in rheumatoid arthritis patients, including increased body weight and dyslipidaemia [171], suggesting that under steady state conditions IL6 is essential for a proper control of metabolic functions [153].

Accordingly, a mounting body of evidence from animal studies indicates that IL6 is a crucial homeostatic regulator of energy intake and expenditure as well as nutrient metabolism [156]. Global IL6 deficient mice develop a late-onset obese phenotype [172], due to decreased EE and muscle fatty acid oxidation [173,174,175], as well as systemic IR, liver inflammation and hepatosteatosis when fed HFD [176]. A subsequent study [177] in mice with specific *IL6RA* deletion in hepatocytes revealed that hepatic IL6 classical signalling essentially contributes to maintain local and systemic insulin sensitivity by facilitating glucose disposal in muscle and adipose tissue. Moreover, classical IL6 signalling in hepatocytes and macrophages confers protection against systemic inflammation by inhibiting TNFA secretion by Kupfer cells (i.e., hepatic resident macrophages) and inducing an IL4 mediated shift in macrophage polarization [178,179]. Additionally, exercised-muscle [180] and adipose tissue [181] derived-IL6 have been also shown to promote insulin secretion via enhanced intestinal and pancreatic glucagon-like peptide 1 (GLP1) production.

Notably, under physiological conditions IL6 is not only a peripherally produced and acting cytokine. IL6 is found in the CNS in health and disease, with cellular sources being glial cells and neurons [155,164,182]. As previously mentioned, exercise is a potent inducer of circulating IL6 levels arising from the contracting skeletal muscle [183] but under such stimulus *IL6* hypothalamic expression also increases at least in rodents [184]. Several studies have explored the effect of centrally acting IL6 on energy and metabolic homeostasis using different approaches: (1) icv IL6 administration [172,184,185] or adenoviral gene transfer [186]; (2) glial-specific induced overexpression or depletion [187,188] and (3) CNS blockage of neuronal IL6 classic or trans-signalling [189,190]. Overall, these studies strongly support a role for IL6 as a homeostatic regulator of body mass, composition and metabolism by reducing food intake and/or increasing EE, ameliorating DIO hypothalamic inflammation as well as central and peripheral IR. Proposed inducers of IL6 and its IL6RA mediated anorexigenic action at the hypothalamus and/or hindbrain included: exercise, GLP1 and the pro-inflammatory cytokine IL1. However, these results have been partially challenged by a recent report showing that blocking trans- but not classic signalling, in the CNS abrogates the ability of IL6 to suppress feeding [189].

#### 3.3.3. IL6 and Thermogenic Function of Adipose Tissues

The critical thermogenic properties of IL6 in the febrile response mounted upon peripheral or central immune challenge have been known for decades [191]. Thus, mice with global deletion of the *IL6* gene do not develop fever upon peripheral IL1 or turpentine administration [192,193]. But it was not until recently that the mechanism by which IL6 mediates fever was clarified. By cellular specific *IL6RA* knockout models, Eskilsson et al. [194] demonstrated that IL6 binding to IL6RA on brain endothelial but not neuronal cells triggers STAT3-induced hypothalamic expression of the prostaglandin synthesizing enzyme cyclooxygenase 2 (*COX2*) and subsequently increases core body temperature.

However, there are several indications that IL6 can be of importance for thermoregulatory and thermogenic control under steady state conditions (i.e., in absence of inflammation). As previously mentioned Wallenius et al. [172,185] reported that icv IL6 administration to rats at physiological doses increased EE without affecting caloric intake or locomotor activity. Indeed, hypothalamic direct adenoviral *IL6* gene delivery suppressed weight gain and visceral adiposity through sympathetically mediated induction of UCP1 protein in BAT, as proven by suppressive effects of tissue denervation [186]. Conversely, *IL6* knockout mice display a blunted increase in EE in response to cold or stress challenge and a lower core temperature than control littermates under thermoneutral or cold ambient conditions, suggesting an impairment of adaptive thermogenesis [173]. In agreement with a central IL6-SNS-mediated action on EE and thermogenesis the same report showed indications of a decreased sympathetic outflow in *IL6* null mice irrespective of gender. Thus, IL6 deficient mice exhibited decreased NE levels after stress challenge but similar increases in EE to those found in control mice after peripheral NE treatment. In line with these findings, lower rectal or core body temperatures than controls have been reported in other mouse models with tissue specific IL6 [195] or *IL6RA* deletion [196] (muscle and CNS) when maintained at warm or cold environments.

Strikingly, rodent and human studies have provided evidence of beneficial and deleterious roles of IL6 mediated activation of WAT browning. Exercise and cold induced UCP1 protein expression in scWAT in mice seems to be IL6 mediated as *IL6* knockout mice show blunted or reduced increases of this uncoupler protein under both stimuli, while its mRNA levels were found to be decreased or normal in relation to control animals [197]. Conversely, IL6 has been shown to induce and sustain WAT browning, aggravating the hypermetabolism observed in conditions of chronic adrenergic and inflammatory stress such as cancer cachexia [30] and severe burn injury [31,198]. Thus, blocking IL6 production by nonsteroidal anti-inflammatory drugs or IL6 neutralizing monoclonal antibody in a mouse model of cancer associated cachexia reduces the severity of wasting and suppresses the browning capacity of subcutaneous WAT. Finally, proposed mechanisms of the browning related burn injury includes: a central stimulatory action of the increased IL6 systemic levels on peripheral production of catecholamines and WAT lipolysis [31], or a direct action of this cytokine on adipose tissue macrophage polarization [199]. Whatever the underlying biological mechanism, disproportionate recruitment and activation of beige adipocytes in WAT of burn patients converts this tissue in an undesirable energy sparing source that might contribute to their adverse outcomes [29].

### 3.4. IL15, a Controversial Myokine in Mitochondrial Function and Thermoregulation

#### 3.4.1. IL15 and IL15/IL15RA Complex

IL15 is a pleiotropic cytokine, functionally and structurally classified as a partial mimetic of the in vitro IL2 activity and a component of the 4-α-helix bundle cytokine family [200]. Thus, IL15 is involved in the proliferation and effector functions of NK and CD8^+^ T cells [200,201,202], while opposing to IL2, no activation-induced cell death or Treg expansion and activation has been reported for IL15 [203,204]. The IL15 heterotrimeric receptor is composed of a specific subunit α (IL15A), that upon high affinity intracellular ligand-binding and exporting to the cell surface can recruit the β and γ chains of the IL2 receptor (IL2RB and IL2RG) at the plasma membrane, or be cleaved after trans-presentation to neighbouring cells displaying the IL2R signalling chains [201,205]. Though, IL15 might also act as a conventional soluble cytokine on cells that express all the receptor subunits, complexation with IL15A highly potentiates its secretion, stability and bioactivity [206,207]. Consequently, complexed IL15 has been longer considered its dominant state in human and mice serum [208]. Additionally, multiple isoforms of IL15A have been reported to either potentiate or inhibit the effects of IL15 [209]. Nevertheless, a recent study has shown that, despite short half-living, an extensive proportion IL15 in mouse serum resides in the free state, which argues against cleavage of membrane-bound IL15/IL15RA as the solely mechanism for IL15 secretion [210].

#### 3.4.2. IL15 and Energy and Metabolic Homeostasis 

The broad range of *IL15* and *IL15A* expression in non-lymphoid tissues including but not limited to, skeletal muscle, gut, adipose tissue, liver and brain (Gene accession ID: 16168 and 16169) pointed to multifaceted actions for this cytokine in vivo [211,212,213]. Initially, IL15 attracted the scientific interest due to its potential role as anabolic cytokine modulating muscle mass [214,215]. Soon after, studies in human subjects and laboratory mice suggested that IL15 acted as a beneficial metabolic factor abundantly expressed by the skeletal muscle [216,217,218,219] and acutely increased in circulation following exercise [220,221,222]. However, this paradigm has been recently challenged by others [223,224,225]. Consequently, a plethora of evidence supported the envision of IL15 as a myokine with potential to counteract obesity and T2DM (reviewed in [211,212,213,226]). Reported IL15 actions in this context include: (1) reduced lipid uptake and synthesis and increased FAs mobilization and oxidation in WAT; (2) amelioration of overall IR by enhanced glucose transport and utilization in muscle and decreased gluconeogenesis in liver; (3) enhanced mitochondrial activity and FAs oxidative capacity in muscle fibres; and (4) decreased lipid intestinal absorption.

Global and muscle specific IL15 transgenic overexpressing (*IL15* Tg and HSA-IL2SP-IL15) and gene-knockout (*IL15* KO) mice, respectively showed a lean and obese mature onset phenotype when reared in a normal chow [227,228,229]. The HSA-IL2SP-IL15 mice displayed constitutively elevated serum IL15 levels along with increased EE and locomotor activity, muscle performance and oxidative metabolism [222,230]. This mouse model was also resistant to diet induced obesity and inflammation, as measured by serum levels of IL6, TNFA and IL1 [231]. These observations together with the negative correlations between abdominal fat mass and circulating IL15 in humans and muscle derived IL15Tg mice, lead to the proposal of a muscle-AT endocrine-axis regulating body composition and insulin sensitivity through induction of lipolysis [232,233]. 

However, this notion is not exempt of controversy. Firstly, whether and to what extent muscle can be considered as a physiologically relevant source of IL15 in human circulation is a matter of discussion [225,234]. Pharmacological and transgenic rodent studies used supraphysiological doses of IL15 not seen in humans [221,232,234]. Indeed, global IL15 Tg mice develop fatal lymphocytic leukaemia, precluding its suitability as a model of the IL15 functions in obesity [235]. Microdialysis studies in obese and lean subjects with similar serum IL15 levels revealed that, as it expands sc WAT produces increasing amounts of IL15 and shows a higher lipolytic rate, while its lower muscle secretion remained constant. Indeed, locally perfused IL15 induces lipolysis in lean sWAT at a rate correlated with its interstitial content [225]. These data suggest an IL15 autocrine/paracrine role rather than the proposed endocrine action on body composition. To solve this issue, further studies on the contribution of both IL15 sources to its increased circulating levels and lipolytic rate in exercise conditions are warranted.

Secondly, in clear contrast to the effects of whole body and muscle IL15 over-secretion, global deletion of *IL15A* in mice also lead to reduced body and fat mass as well as increased caloric expenditure, with independence of diet intake and its caloric content [236,237]. Of note, both mouse strains shared a hyperactive and fast muscle fatigue resistant phenotype associated to a switch towards a FA oxidative molecular signature in slow and mixed myofibers (i.e., increased levels of mitochondrial associated transcription factor-, PPARD and its coactivator, PPARGC1B) [209,229,231]. Conversely, some differences exist, while IL15A KO mice are glucose intolerant and insulin resistant, independent of the diet and age [237], *IL15* over-expression in muscle increases insulin sensitivity in HFD fed mice [231]. To complicate matters further, the previously reported obese *IL15* KO mice [227,228] have recently been shown to resist diet induced overall AT accumulation, visceral AT inflammation and IR, through enhanced EE (discussed in detail below) [238].

Overall, these observations pose doubts regarding whether this cytokine exerts pathogenic or beneficial metabolic effects (i.e., promotion of EE, muscle performance and insulin sensitivity) and warrant further investigation. Various hypothesis has been raised to reconcile or rebut these findings: (1) increases in circulating free IL15 [210,237] and an IL15 induction of pro-inflammatory cytokines from resident immune cells in AT of *IL15A* KO mice [238,239] and (2) differences in the composition of gut microbiota between mouse strains [228,238].

#### 3.4.3. IL15 and Thermogenic Function of Adipose Tissues

When it comes to the putative roles of IL15 in thermogenesis, BAT function and browning of WAT, controversy persists. Former studies reported that chronic IL15 administration in rats increased whole-body FA oxidation and upregulated BAT gene expression levels of the thermogenic proteins UCP1 and UCP3, as well of those of PPARD and PPARGC1A and its targets, the FA mitochondrial transporters CPT1 and CPT2 [240]. More recently, untargeted gene transfer in DIO mice transiently increasing serum levels of complexed IL15:IL15A was reported to induce similar effects in BAT, together with a significant induction of *PPARGC1A* and *PPARGC1B* mRNA levels in scWAT. Though from these results one might infer an IL15 mediated induction of BAT thermogenesis but, unfortunately no confirmatory EE measurements were performed in those studies [240,241]. Muscle *IL15* Tg mice exhibited no induction of UCP1 or other markers of brown/beige fat such as PRDM16 (beige fat phenotype inducer) in several adipose tissue depots. As muscle was considered primary site for FAs utilization in HFD fed *IL15RA* KO mice, no data are available from their BAT or WAT molecular phenotype.

As above mentioned, both muscle specific gain and global loss of function mutant mice showed elevated levels of whole-body EE; but postprandial increases in core body temperature, consistent with adaptive thermogenesis and independent of the fed diet, were only detected in null *IL15RA* [236,237] and *IL15* mice [238]. The higher hypothalamic orexin and transient receptor potential vanilloid 4 cation channel (*TPRV4*) gene expression observed in low fat diet (LFD) fed *IL15RA* deficient mice is consistent with a central role of this cytokine in thermoregulation and inhibition of BAT thermogenic activity. Likewise, BAT from HFD fed null *IL15* mice tend to a higher FAs uptake than WT controls along with enhanced gene expression of: *COX-IV* (circadian component of the mitochondrial respiratory chain), mitochondrial transcription factors (*PPARG*, *NRF1* and *TFAM*) and thermogenesis markers (*UCP1*, *EVOLV3* and *CIDEA*). A similar transcriptional pattern, including upregulation of PRDM16, was observed in this model upon stimulation of WAT browning program by cold exposure or sympathomimetics. In addition, lack of endogenous *IL15* in primary in vitro cultures of differentiated brown and white adipocytes, markedly enhanced basal cells oxygen consumption rates (OCR) that was reversed by IL15 treatment. Finally, the same study provided in vitro indications of an IL15 inhibitory action on lipid accumulation and thermogenesis (decreased *UCP1* and *CD36* gene expression) in human beige cells. Overall these findings suggest that during conditions of lipid overload, IL15 impedes the white to beige AT phenotypic switch and impairs the capacity of BAT and beige cells to utilize circulating FAs, activate mitochondrial oxidative phosphorylation and uncoupling. Thus, by perpetuating inflammation and lipid spill-over, IL15 in combination with other proinflammatory factors might cause IR and inhibition of adaptive thermogenesis.

To date, there is no plausible explanation for these paradoxical findings but it is tempting to speculate that the tissue inflammatory microenvironment might mark the limit between the beneficial and pathological effects of IL15. Adiponectin to leptin ratio has been proposed as a promising index to estimate adipose tissue dysfunction and IR and its negatively correlated to markers of low-grade inflammation. While, adiponectin secretion from 3T3-L1 adipocytes is stimulated by IL15, muscle derived *IL15* Tg mice exhibited reduced circulating leptin concentrations with a concerted loss of intra-abdominal fat, which could explain the beneficial effect of IL15 on insulin sensitivity in this model. Additionally, IL15 has been shown to either suppress or counteract the negative effects of TNFA in human myogenesis, myotube development and function in mouse models of sarcopenia and cachexia. Whether the proposed IL15 inhibitory role of WAT browning in obesity could turn into a positive effect in the hypermetabolic context of cachexia remains to be elucidated.

### 3.5. IL18/IL18R1, a Complex Immune System in the Control of BAT and Beige Thermogenesis

#### 3.5.1. IL18 Production and Signalling

As IL33, IL18 is a member of the IL1 family of cytokines, which participates in immune responses by co-stimulating INFG production, Th1 cell proliferation and activation of NK cells [242]. *IL18* (Gene ID: 16173) is constitutively expressed by most cells in healthy humans and animals as a nuclear inactive precursor, which requires cleavage by the protease caspase 1 (CASP1) into a mature active cytokine to be secreted [243]. CASP1 itself is synthesized as a zymogen, which needs to be oligomerized and activated in a cytoplasmic protein complex known as the inflammasome [244]. Complexation of the inflammasome is triggered through recognition by its components, the NOD-like receptor (NLR) sensor molecules, of substances derived during infectious processes, tissue damage or metabolic imbalances. Several inflammasomes formed by NRLs and the pyrin domain (P) containing adaptor protein (ASC) with the ability of recruit pro-CASPases have been described but NRLP3 and NRLP1 have been mainly associated with mature IL18 production [245]. Thanks to this complex system, immune and non-immune cells in several metabolic organs/tissues, such as brain, liver, skeletal muscle and specially ATs can release active IL18 in response to infectious, inflammatory and metabolic cellular stress [246].

IL18 signals through a heterodimeric complex composed of a ligand binding subunit (IL18R1) and a signal-transducing chain or accessory protein (IL18RAP) [247]. Both chains are essential for MYD88 and IRAK recruitment, subsequent translocation to the nucleus of NFKB and pro-inflammatory gene transcription. Energy metabolism signalling pathways are also triggered by this cytokine including: STAT3, as well as mitogen-activated protein, phosphoinositide-3 and AMP-activated protein kinases (MAPK, PI3K and AMPK) [248]. Three naturally occurring inhibitors have been proposed as titters of IL18 activity [242,249]: Its high affinity soluble binding protein (IL18BP) and two splice variants of its receptor subunits, claimed to act as decoy receptors.

#### 3.5.2. IL18 and Energy and Metabolic Homeostasis

As in the case of IL6, elevated circulating IL18 serum levels were found in obese and T2DM patients, reduced after weight loss and were postulated as risk predictors for metabolic syndrome development before detection of obesity and IR [250,251]. Again resembling the case of IL6 but also IL15, former studies by Netea [252] and Zorrilla et al. [253,254] showed that *IL8* null mice developed mature onset obesity, irrespective of the diet fed and gender, not only due to hyperphagia and hypoactivity but also disturbances in peripheral nutrient metabolism. Thus, IL18 deficiency decreased insulin sensitivity and increased fuel-efficiency, whereas central and/or peripheral IL18 administration counteracted these effects. Moreover, mice lacking IL18R1 and overexpressing IL18BP also displayed insulin resistance, hyperglycaemia and obesity. Conversely, skeletal muscle *IL18*-overexpression induces resistance to dietary obesity in mice through induction of AMPK signalling and lipid oxidation, which can then balance lipid accumulation on a HFD [248]. The apparent contradiction between human and mice data was partially solved by Zilverschoon et al. [255], who reported that leukocytes from obese and T2DMs patients exhibited defective IL18-mediated signalling due to decreased expression of *IL18RA* and *IL18RAP*, which render them resistant to the IL18 catabolic action.

Murphy et al. [245] by using several lines transgenic mice have recently provided crucial insights into the trigger stimuli, cellular and tissue source of increased circulating IL18 during obesity and T2DM. Previous reports had shown that serum IL18 was increased in humans shortly after a high-fat meal but not after a high-carbohydrate meal [256]. Thereby, Murphy et al. [245] demonstrated that the innate immune sensor NRLP1 is able to detect increases in the caloric content of the diet (either enriched in fat or in proteins) and activate the inflammasome mediated production of IL18 preventing obesity and metabolic syndrome, as shown in mice overexpressing *NRLP1*. The mechanism proposed for such effects, implied peripheral activation of AMPK, increased glucose uptake and lipid oxidation, leading to defective lipid accumulation in the AT and amelioration of IR.

#### 3.5.3. IL18 and Thermogenic Function of Adipose Tissues

In contrast to the strong pyrogenic activity of IL1 and IL6, IL18 is only able to induce fever at higher concentrations, suggesting that this cytokine do not mediate infection driven thermoregulation [247]. However, as previously discussed mice with global deficiency of IL18 and IL18R1 have been reported to exhibit decreased EE as causative factor of their metabolic phenotype as well as disturbances in FFAs oxidation at the level of the muscle and ATs. These observations and the parallelism between IL6 and IL18 actions in muscle tissue led us to hypothesize that IL18 could also play a role in AT plasticity and thermogenesis. Thus, we carefully analysed the phenotype of IL8 and IL18R1 deficient mice under different thermogenic stimuli: HFD and short and prolonged cold exposure [246]. Strikingly, we found opposite responses in both strains of mice. Cold exposed *IL18R1* KO mice were protected against acute body temperature drop. Inguinal fat pads from *IL18RI* null mice displayed a more brown-like structure, alternative macrophage activation and thermogenic gene expression (*UCP1*, *PRDM16*, *PPARGA*) than those of WT controls. Conversely, *IL18* KO mice were hypothermic when cold challenged and the browning of their scWAT was almost blunted as shown by histological, protein and gene expression analysis. Additionally, *IL18R1* KO mice showed decreased energy EE when fed an LFD and acute HFD challenge blunted this effect. In the long term, feeding with an HFD also demonstrated divergences between *IL18* and *IL18R1* null mice. Mice deficient in the ligand were extremely prone to DIO, while deletion of the receptor conferred resistance to its obesogenic effect. In both cases *UCP1* mRNA levels in sc WAT paralleled these phenotypes. Overall, these results suggest that IL18 can induce BAT activation and browning of sc WAT but the mechanism responsible of these actions might be related to a more complex ligand-receptor interaction.

### 3.6. IL10/IL10RA, an Anti-Inflammatory Brake of Thermogenic Gene Expression and EE 

#### 3.6.1. IL10 Production and Signalling

Last but not least, the classical anti-inflammatory cytokine IL10 has been recently incorporated to the growing list of interleukins with actions on brown and beige fat function [257]. This type 2 cytokine, known by its key role in avoiding inflammatory and autoimmune pathologies [258,259], gives name and belongs to a family of interleukins such as IL19, IL20, IL22, IL26 and IL29. All of them share similarities in receptor structure and gene organization but display diverse biological activities ranging from immune suppression and antitumoral activity, to enhanced antibacterial and antiviral immunity. [260]. Several adaptive and innate immune cells produce IL10 including B and T cells and Tregs, as well as macrophages, dendritic cells (DCs) and eosinophils [257,259]. As for other previously discussed interleukins both the timing and site of its production are clear determinants of the magnitude and identity of IL10 actions. However, major immune functions described for this cytokine are: (1) limitation of Th1 and Th2 responses of macrophages and DCs; (2) enhanced Treg differentiation; (3) inhibition of CD4^+^ T cells proliferation and (4) under certain conditions promote B-cell activation and NK-cell proliferation [258]. Once produced and secreted, IL10 binds to membrane anchored subunit α of its receptor IL10RA and forms a dimeric receptor with the subunit β (IL10RB, an accessory protein) [261], that leads to activation of JAK1 and tyrosine kinase 2 (TYK2) and subsequently of STAT3 [262]. IL10 anti-inflammatory activity is believed to be primarily due to STAT3 dependent-repression of pro-inflammatory cytokines and chemokines gene transcription [257,263].

The expression of this potent anti-inflammatory cytokine and its ligand binding receptor subunit *IL10RA* is primarily restricted to hematopoietic cells (Gene accession number IDs: 16153 and 16154). However, one or the other can also be produced by non-immune cell types such as epithelial cells, keratinocytes and placental cytotrophoblasts, more often in an induced rather than a constitutive manner. Conversely, *IL10RB* is almost ubiquitously expressed under steady state conditions (accession number ID: 16155). Thereby, any stimulus capable of activating *IL10RA* production might render most cells responsive to IL10 (extensively reviewed in [261]). 

#### 3.6.2. IL10 and Energy and Metabolic Homeostasis

Human and rodent studies have also provided contradictory results regarding the role of this cytokine in the regulation of body composition, energy balance and metabolic function. Circulating IL10 levels have been shown to be either negatively [264,265], positively [266] or not correlated [267] with obesity and fat mass content in humans. The reason of this discrepancy might be related to: (1) divergent production of IL10 from its AT cellular sources (increased in adipocytes [268] and decreased in infiltrated ATMs [92]) and (2) the influence of gender (due to modulatory actions of oestrogens [265,269]) or ethnicity of subjects studied [270]. However, at least in human studies, there was a general agreement in the literature regarding the association between low systemic levels [264,265,266] or decreased production capacity [271] of IL10 with indexes of both the metabolic syndrome and T2DM. Conversely, exercise a well-known insulin sensitizer has been proven to promote in an IL6 dependent manner the appearance in circulation of IL10 and other anti-inflammatory cytokines such as IL1RA (the IL1 endogenous antagonist), while inhibiting TNFA production [272]. Altogether these results led to the hypothesis that IL10, acting individually or jointly with IL6, could prevent or ameliorate obesity as well as associated IR and lipid disturbances [184]. 

Pharmacological and/or transgenic approaches to block or enhance IL10 whole-body or tissue specific production (mainly in muscle and liver or at CNS level) in rodent models have either confirmed or dismissed such envision. Overall, former studies using HFD-fed *IL10* null mice (from 4 to 36 weeks) suggested a time dependent effect of lack of *IL10* on body weight, adiposity and overall insulin sensitivity [273,274,275,276]. Lower increases in obesity, adiposity and insulin resistance were found in IL10 deficient than in WT controls after 6 months on an HFD. Meanwhile, shorter dieting periods in this mouse model initially increased hepatic TG content and thereafter were related to ameliorated hepatic steatosis with unaltered body weight, energy intake or expenditure and IR. Strikingly, in a thorough recent study Rajbhandari et al. [257] have shown that *IL10* KO mice develop a mature onset lean phenotype (8 months of age) characterized by decreased fat mass, improved glucose tolerance as well as protection against aging-induced hepatic steatosis, hypertriglyceridemia and AT inflammation. This anti-obesity effects of lack of *IL10* are related to a heightened EE despite increases in LFD food intake were concomitantly found. In the same study, *IL10* null mice were also shown to be protected against DIO (6 weeks on HFD) and its associated metabolic disturbances by increased EE and thermogenesis (discussed in detailed below), in absence of overt symptoms of inflammatory disease or reduced food intake (similar to that of littermate controls). These contradictory results between studies might be associated to differences of genetic background of *IL10* null mice, husbandry and housing conditions (including diet type and vivarium conditions) or the influence of closely related cytokines such as IL6.

To further complicate matters, IL10 or IL10RA knockdown by neutralizing antibody or an antisense oligonucleotide (ASO) peripheral injection as well as IL10 exogenous administration, hydrodynamic gene delivery or muscle specific overexpression in mice have rendered quite opposite outcomes. Cintra et al. [277] showed that hepatic specific IL10 inhibition for 5 days with ASO or IL10-antibodies downregulated hepatic insulin signal transduction in association with increased local inflammation as well as gene expression of gluconeogenic and lipid synthesis related enzymes. In contrast, Rajbhandari et al. [257] have shown that *IL10RA* knockdown specifically in AT with ASO treatment for 3 weeks markedly reduced body and fat mass without obvious systemic inflammation. Conversely, IL10 acute peripheral treatment [278] and muscle specific overexpression in mice under LFD or 3 to 16 weeks of HFD, respectively, were shown to improve hepatic and/or muscle insulin action and glucose turnover while body weight remained unchanged [279] or even increased [280]. Finally, Gao et al. [281] reported that tail vein *IL10* gene delivery by plasmid injection for 7 weeks, rising serum and IL10 hepatic serum levels, was able to counteract DIO by blocking adipocyte hypertrophy, ectopic fat accumulation (at the liver and pancreas). These effects were not mediated by decreased energy intake and EE measurements were not reported. Whole-body metabolic improvements were achieved by blocking ATMs infiltration, fat inflammation and subsequent adipocyte death as well as by preventing hyperinsulinemia, maintaining insulin sensitivity and preventing glucose intolerance. Overall these results suggest that as in the case of IL6, IL10 seems to act as pleiotropic cytokine that, depending on the inflammatory milieu, its cell source and tissue targets, exerts dose and time dependent effects on body weight and composition, energy balance and metabolic function. Indeed, Ropelle et al. [184] reported that under chronic over nutrition conditions, exercise-induced hypothalamic increases in *IL6* and *IL10* expression can restore energy homeostasis by reducing food intake. Mechanistically, the anti-obesity effect of these cytokines was mediated by inhibition of IKKβ activation and endoplasmatic reticulum stress leading to restoration of central insulin and leptin sensitivity.

#### 3.6.3. IL10 and Thermogenic Function of Adipose Tissues

As previously discussed, results published early this year [257] demonstrated that *IL10* deficiency in mice promotes whole-body EE and increased mitochondrial activity in scWAT, as measured by basal and stimulated OCRs (basal and maximal respiration). In agreement, *IL10* null scWAT gene expression profile closely resembled that of BAT than did that of WT mice, including increased *UCP1* mRNA and protein levels. Upregulation of thermogenic gene expression was confined to this tissue since no difference between genotypes were found in BAT and were attenuated when mice were housed at thermoneutrality. However, core temperature and catecholamine levels were not affected by lack of *IL10*, suggesting that this cytokine acts antagonizing WAT adrenergic tone by downstream mechanisms. Bone marrow (BM) was identified as the source of IL10 activating fat browning thanks to BM transplantation studies in irradiated control and *IL10* null mice. Additionally, *IL10RA* mRNA was found to be abundantly expressed in mature adipocytes and upregulated by HFD feeding, obesity, aging and PPARG activation. Further confirming the scWAT as a target of IL10, *IL10RA* knock-down or out by specific ASO treatment or adenoviral gene silencing markedly increased thermogenic gene expression in mouse scWAT. Finally, authors demonstrated that this cytokine acts as a specific repressor of the thermogenic gene program rather than a general inhibitor of adipocyte transcription. Thereby, IL10 exerts its anti-thermogenic transcriptional effects by altering chromatin accessibility and recruitment of transcription factors (ATF and CEBPB) to regulatory gene regions. Taking together, these results and the key immunomodulatory role of IL10 suggest that the IL10/IL10RA might act as a brake to limit fat burning and preserve fuel supplies in conditions of acute energy demands as infectious states but also in obesity and aging. The beauty of this proposal resides in the fact that unlike IL1 [282] and TNFA [283] (acting specifically in BAT), IL10 is able to block the thermogenic response by inhibiting browning of scWAT.

## 4. Conclusions and Future Perspectives

Decades of research have revealed that the role of AT extends far beyond its function as simple energy reservoir. Instead, its anatomical compartments/depots show a clear developmental, cellular and functional heterogeneity, conferring to this organ a crucial metabolic relevance. Energy-burning BAT and beige ATs have emerged as key endogenous defences to tackle the global threat of obesity and T2DM. Type 2 immune cell (macrophages, eosinophils and ILC2s) and their signalling mediators, cytokines, have been proposed as positive actors in the local control of BAT activation and WAT browning. However, some controversies and contradictory results have been recently reported regarding mechanism behind the role played by this immune-AT axis. In this review, we have tried to highlight and discuss major strengths and flaws of this emerging paradigm. Taking as a whole, present data suggest that a preferential anti-inflammatory phenotype among the resident and infiltrated immune cell subpopulations and subsequent secretion of related interleukins contribute to: (1) support mature beige adipocytes development from their multipotential precursors; (2) modulate local adrenergic input to WAT through yet incompletely defined mechanisms and (3) limit AT inflammation and IR and favour FFA oxidation thereby creating a favourable micro-environment for classical thermogenic activation and recruitment of beige adipocytes. Further research is warranted to clearly delineate the biological relevance of each immune cell type in this process and identify new cytokines secreted also by parenchymal and stromal cells to support energy production and thermoregulation by ATs. This is a quite challenging issue taking into account that ideally, variations in age, gender and type of diet should be considered in the different experimental paradigms to be assessed.

Of note, it is important to keep in mind that as for almost biological responses, the functional outcomes depend on the balance between benefits and harms. Thus, some of these cytokines have also been identified as triggers of the deleterious effects of browning in hypermetabolic states such as cachexia and burn injury. A precis mechanistic insight regarding the cell sources and targets, as well as signalling pathways activated in these pathological situations would help to inform future therapies. Aside their local action at the ATs, various interleukins (such as IL6 and IL15) have been proposed as central regulators of energy and nutrient metabolism balance by targeting CNS nuclei involved in the thermogenic efferent pathway controlling BAT function and WAT browning. Given the paucity of data regarding the putative role of these immune related factors in this scenario, future research efforts in this line would also be of great interest.

## Figures and Tables

**Table 1:** Summary of cellular transfer, transgenic or pharmacological approaches targeting cytokine signalling with effects on BAT activity and beige fat recruitment.

Reference	Interventional Approach	Cell/Cytokine/Intracellular Mediator	Rodent Model (Genetic Background)	Age (week)	External Cue (T °C, Diet, Treatment)	Gender	Effects on EE, Thermogenesis and Metabolic Homeostasis
Nguyen, 2011	Global knockout	IL4/IL13 STAT6	BALB/cJ BALB/cJ or C5BL6J	8–12	4 °C, 6 h	male	Decreased weight loss Cold-induced hypothermia Decreased BAT thermogenic gene expression Exhausted lipid stores in BAT Decreased serum FFA Blunted M2-like markers in BAT and WAT
	Conditional knockout, myeloid-specific	IL4RA	BALB/cJ IL4RαL/LLysMCre				
	Global knockout	IL4/IL13	BALB/cJ		4 °C, 6 h Acute β3-agonist treatment		Normalized weight loss Increased EE Increased core body temperature Increased thermogenic gene expression Increased lipid storage in BAT
	Global deletion, clodronate liposomes treatment	Macrophages			4 °C, 6 h		Cold-induced hypothermia Decreased BAT thermogenic gene expression Blunted M2-like markers in BAT and WAT
Qiu, 2014	Global knockout	IL4/IL13 STAT6 IL4RA	BALB/cJ	12	4 °C, 48 h	male	Decreased cold induced EE (VO2) (STAT6 and IL4RA KO) Cold-induced hypothermia Impaired browning Reduced sc WAT thermogenic gene expression Decreased scWAT oxygen consumption (IL4/IL13 KO)
	Eosinophil deficient 4get/ΔdblGata mice	Eosinophils					Decreased cold induced EE (VO2) Impaired browning Reduced sc WAT thermogenic gene expression
	Global knockout	CCR2					Decreased cold-induced ATM recruitment Decreased cold induced EE (VO2) Impaired browning Reduced sc WAT thermogenic gene expression
	IL4 i.p. treatment (IL4 complexed)	-	DIO C57BL6/J		HFD 10 weeks 30 °C IL4 treatment 14 days		Decreased body weight Decreased fat mass Improved insulin sensitivity Increased browning
Brestoff, 2015	Global knockout	IL33	C57BL6/J	7	LFD 12 weeks	male	Increased body weight Increased fat mass Insulin resistance Decreased beige adipocytes in scWAT Decreased ILC2s content in scWAT
	IL33 i.p. treatment	-		8	LFD 12 weeks IL33 treatment 7 days		Decreased fat mass Increased EE Increased browning in scWAT
					HFD and IL33 treatment 4 weeks		Counteracts DIO Abrogates glucose intolerance Increases ILC2s and Treg content in WAT
	adoptively transferred congenic ILC2		ILC2-deficient Rag 2 mice		IL33 treatment 7 days		Increased UCP1 protein in iWAT Increased iWAT browning dependent on ILC2s
Lee, 2015	IL33 i.p. treatment IL13 i.p. treatment IL4 i.p. treatment		C57BL6/J or IL5^Red5^^/+^, R5 BALB/cJ	8–12	Cytokine treatment 8 days 30 °C	male	Increased browning of scWAT Elicited beige progenitors (IL33, R5 mice) Increased scWAT UCP1 protein levels Increase cold-induced EE (IL33 treatment)
	Global knockout	IL5 (eosinophil growth factor) Normal IL13 secretion	IL5^Red5^^/Red5^ BALB/cJ		IL33 treatment 8 days 30 °C		Elicited proliferation of beige progenitors
		IL4/IL13 IL4RA	BALB/cJ				Failed to increase proliferation of beige progenitors
	IL4 i.p. treatment (IL4 complexed)		C57BL6/J		30 °C IL4 treatment, 24–48 h		Elicited proliferation of beige progenitors
	Conditional knockout, Progenitor cells specific	IL4RA	IL4RA^f/f^Pdgfra^Cre^				Failed to increase proliferation of beige progenitors
Odegaard, 2016	Global knockout	IL33 IL1R1 (ST2)		Adult: 8–12 Perinatal: 3–4	5 °C 48 h	male and female	Impaired cold-induced iWAT *UCP1* expression Impaired browning Decreased survival in cold
Fisher, 2017	IL4 i.p. treatment Global knockout	None IL4RA	C57BL6/J	12	Daily treatment 14 days Declining T-30–5 °C	male	Unchanged body weight and EE No activation of thermogenic gene program in iWAT
Ding, 2016	IL33 i.p. treatment	-	C57BL6/J	6	HFD 11 weeks IL33 treatment 7 days	male	Restoration of ILC2s and eosinophils content in scWAT Increased UCP1 protein level in scWAT
	ST2 antibody treatment			7	ST2 antibody treatment 4 °C 48 h		Blunted ILC2s and eosinophils recruitment Decreased UCP1 protein levels in WAT
Wallenius, 2002	IL6 icv administration	-	Sprague-Dawley rats		Acute IL6 treatment	male	Increased EE (VO2) Lowers body weight and fat mass Unchanged food intake and activity
Wernstedt, 2003 Wallenius, 2002	Global knockout	IL6	C57BL6/J	8	Cold challenge (6 h 4 °C) Stress challenge (1 h)	male	Spontaneous mature onset obesity Decreased EE in cold and stress Lower body core temperature Decreased NE serum levels
Li, 2002	Adenoviral IL6 gene delivery icv	IL6	Sprague-Dawley rats	-	5 weeks	male	Supressed weight gain and adiposity Increased BAT UCP1 protein levels Blunted by denervation of BAT
Knudsen, 2014	Global knockout	IL6	C57BL6/J	8	Treadmill running 5 weeks or 4 °C, 3 days	male	Reduced sc WAT browning and UCP1 levels Partially reversed by IL6 treatment
	IL6 i.p. treatment	-	C57BL6/J		7 days	male	Increased sc WAT UCP1 levels
Petruzzelli,2014	Transgenic mice with epithelial cell specific overexpression (cancer cachexia)	SOS-F	K5-SOS (skin tumours) C57BL6/J	5	Anti-IL6 Ab BAT denervation		Loss of body weight Fat and muscle wasting Increased UCP1 in sc WAT Increased EE Effects blunted by blocking IL6 or denervation
Patsouris, 2015	Global knockout Burn mice	IL6	C57BL6/J		Burn back by 98 °C for 10 s Evaluation 2 days post-burn		Increase scWAT browning in WT Increased scWAT UCP1 levels Effects blunted in IL6KO mice Effects blunted after propranolol treatment
Almendro, 2008	IL15 i.p. treatment	-	Wistar rats	-	Daily administration for 7 days	male	Decreased WAT and BAT mass Increased BAT *UCP1* gene expression Increased expression of FA oxidation genes
Sun, 2016	Hydrodynamic gene delivery Untargeted overexpression	IL15:IL15A	DIO C57BL6/J	6	10 weeks Administration every 10 days	male	Reduced body weight Reduced adiposity Increased thermogenic markers in BAT and iWAT Improved insulin sensitivity
Lacraz, 2016	Global knockout	IL15	C57BL6/J	4	16 weeks on HFD or 10 °C, 20 h or β3-agonist treatment	male	Resistance to DIO and IR Higher EE than controls Increased expression of genes associated with thermogenesis Elevated basal core temperature Increased BAT activation and iWAT browning in response to cold
Pazos, 2015	Global knockout	IL18	C57BL6/J	8	10 weeks of HFD 4 °C 6 h 4 °C 5 days	male	HFD obesity prone Decreased UCP1 expression in BAT and iWAT Hypothermic after short cold challenge Null browning of scWAT in response to cold
		IL18R1	C57BL6/J		10 weeks of HFD Acute HFD challenge 4 °C 6 h 4 °C 5 days		DIO resistant Increased UCP1 expression in sc WAT Increased EE in response to HFD challenge Maintenance of body temperature in cold Increased browning of scWAT and activation of thermogenic program

BAT: brown adipose tissue; FFA: free fatty acid; WAT: white adipose tissue; EE: energy expenditure; DIO: diet induced obesity; UCP1: uncoupling protein 1; iWAT: inguinal white adipose tissue; scWAT: subcutaneous WAT; VO2: oxygen consumption; NE: norepinephrine; IR: insulin resistance; HFD: high fat diet; ILC2s: innate lymphoid type 2 cells.
